# Biopsy-Proven Melanosis Coli Initially Diagnosed As Candidiasis on Colonoscopy: A Report of Two Cases From North Central Nigeria

**DOI:** 10.7759/cureus.34393

**Published:** 2023-01-30

**Authors:** Kevin N Ezike, Ijeoma A Okwudire-Ejeh, Iliya K Salu, Chidi V Nnabuchi, Michael E Aghahowa, Frank O Ani, Hope M Isaac

**Affiliations:** 1 Anatomic Pathology and Forensic Medicine, Nile University of Nigeria, Abuja, NGA; 2 Anatomic Pathology and Forensic Medicine, Asokoro District Hospital, Abuja, NGA; 3 Surgery, Nile University of Nigeria, Abuja, NGA; 4 Surgery, Asokoro District Hospital, Abuja, NGA; 5 Internal Medicine, Nile University of Nigeria, Abuja, NGA; 6 Obstetrics and Gynaecology, Asokoro District Hospital, Abuja, NGA; 7 Surgery, Trust Charitos Hospital, Abuja, NGA

**Keywords:** melanosis coli, whitish patches, colonoscopy, laxative use, chronic constipation

## Abstract

Melanosis coli is a benign condition, often identified as an incidental finding during colonoscopy, characterized by brown or black pigmentation of the colonic mucosa due to lipofuscin deposition within the cytoplasm of cells. It has been linked to the excessive use of laxatives, particularly those that are anthraquinone-based but also stimulant laxatives and herbal remedies. White patches on colonoscopy in this condition are an extremely rare finding.

We present two cases of 31- and 38-year-old, male Nigerians, with a history of chronic constipation and prolonged stimulant laxative use in whom colonoscopy findings of white patches on the colonic mucosa were confirmed on histology to be melanosis coli.

Melanosis coli should be considered in the differential diagnosis of patients with chronic constipation and/or prolonged use of laxatives or herbal remedies who exhibit mucosal changes on colonoscopy even if these changes are not black or brown discolorations.

## Introduction

Melanosis coli (MC) is a benign condition, often identified incidentally during colonoscopy, characterized by brown or black pigmentation of the colonic mucosa due to lipofuscin deposition within the cytoplasm of cells [1]. This condition, first described in 1830, has been linked to the excessive use of laxatives, particularly those that are anthraquinone-based [1,2]. The pigment deposited is not melanin as the name might suggest, but lipofuscin, proven by electron microscopy and X-ray analyzing methods [3]. Melanosis coli is typically seen in the large intestine and more on the right side [1-5]. The rate of melanosis coli increases with age due to the fact that older persons are more prone to chronic constipation and hence the use of laxatives, and it occurs at the same frequency in males and females [2,3]. Anthraquinones in laxatives are directly toxic to colonic epithelial cells, and they become active substances as they pass the bowel; this causes cell damage and death of the colonic epithelium. The death of the cells creates the dark pigment, which is engulfed by macrophages within the lamina propria [2,6]. The small intestine is usually not affected by the pigmentation in melanosis coli, and this is thought to be due to the absence of anthraquinone receptors in the small bowel and its conversion by colonic bacteria to its active metabolites [7].

The typical presentation of melanosis is a brown or jet-black appearance of the colonic mucosa on endoscopy [1,2,8,9]. We report two cases of melanosis coli that presented, on colonoscopy, as whitish patches on the colonic mucosa and were thought to be candidiasis by the endoscopists; underscoring the need for histological diagnosis for confirmation.

## Case presentation

Case 1

**Figure 1:**
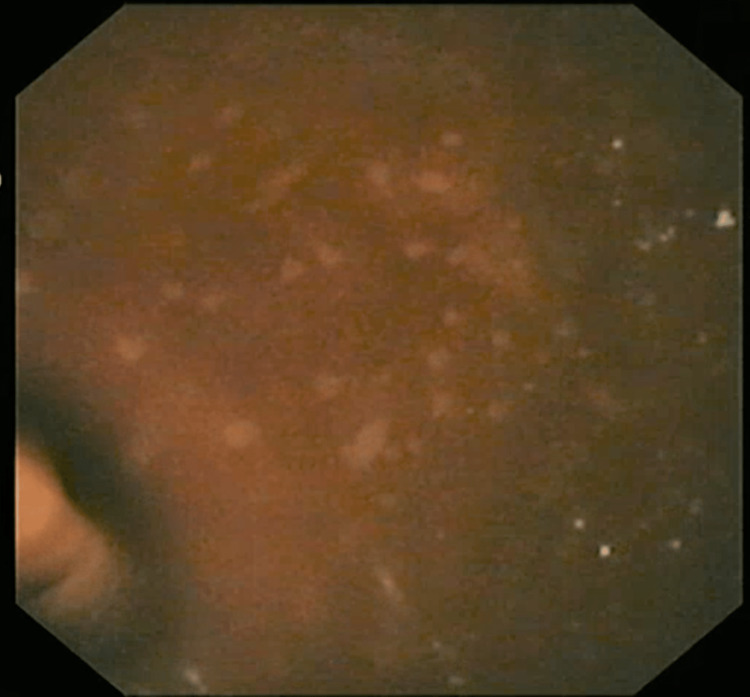
Colonoscopy image (case 1) – note: whitish patches on colonic mucosa

A 31-year-old male presented with recurrent abdominal pain of more than five years duration, and a history of similar duration for the intermittent use of the stimulant laxative, bisacodyl, and the osmotic laxative, lactulose. He had a colonoscopy done, which showed whitish, patchy lesions on the mucosae of the caecum and transverse colon suspected to be candidiasis, and biopsies were taken (Figure 1).

**Figure 2:**
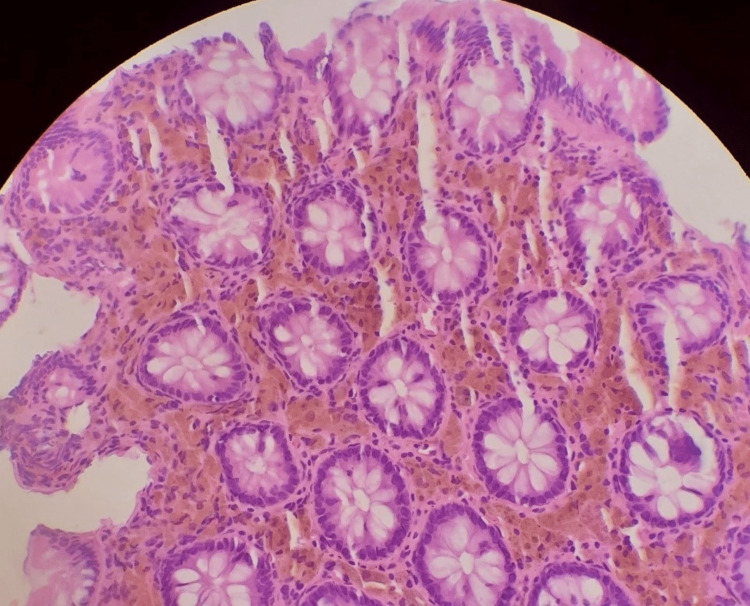
Photomicrograph case 1, H&E x20 – note: pigment-laden macrophages in lamina propria

Histology revealed irregular fragments composed of colonic mucosa with unremarkable epithelium, which overlies lamina propria with quiescent goblet cell-rich glands. Numerous pigment-laden macrophages with golden-brown, granular cytoplasm were seen in the lamina propria. Scanty mixed inflammatory infiltrates and occasional foci of congested, thin-walled vascular channels were also seen. Fungal hyphae and/or spores were not seen. No atypical cells were seen (Figure 2). A diagnosis of melanosis coli was made.

**Figure 3:**
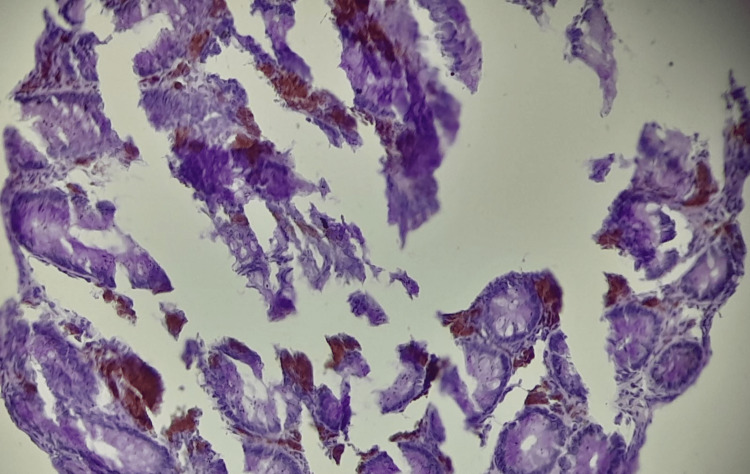
Photomicrograph case 1, PAS x20 – note: uniform purple particles in the lamina propria macrophages PAS: periodic acid-Schiff

**Figure 4:**
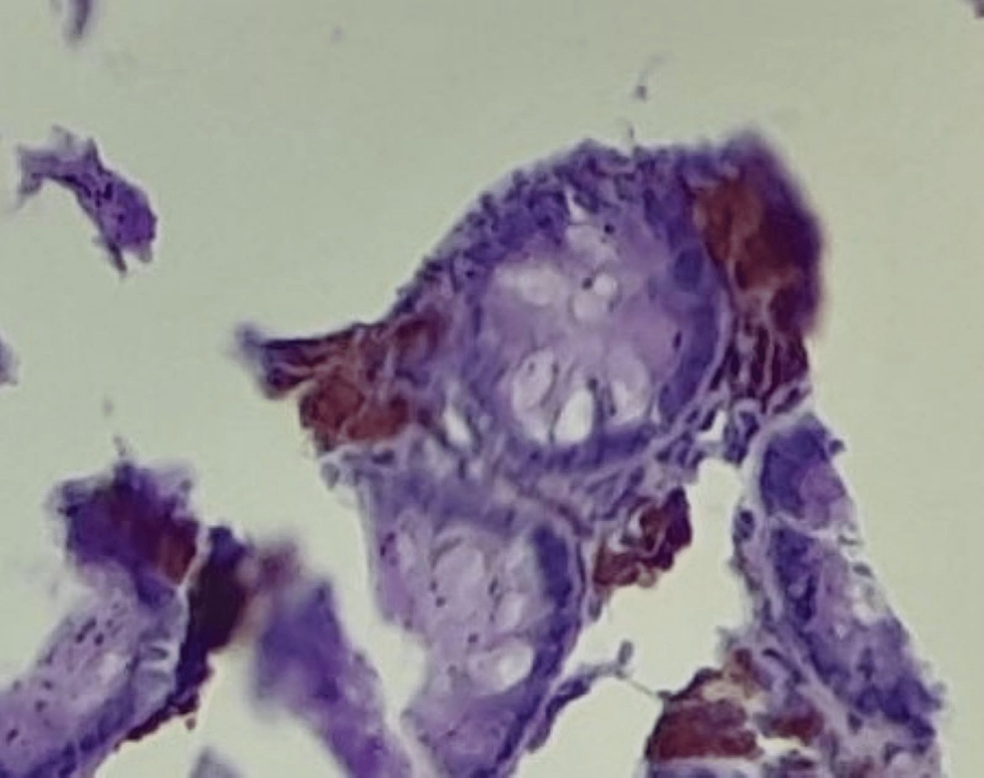
Photomicrograph case 1, PAS x100 (zoom) – note: uniform purple particles in the lamina propria PAS: periodic acid-Schiff

For confirmation, periodic acid-Schiff (PAS) histochemical, and human melanoma black 45 (HMB45) stains were applied to sections from the paraffin wax blocks of the specimen, which stained positive and negative, respectively (Figures 3, 4).

He was counseled on the need to use laxatives sparingly, and to improve the constipation he suffered from by increasing fiber intake.

Case 2

**Figure 5:**
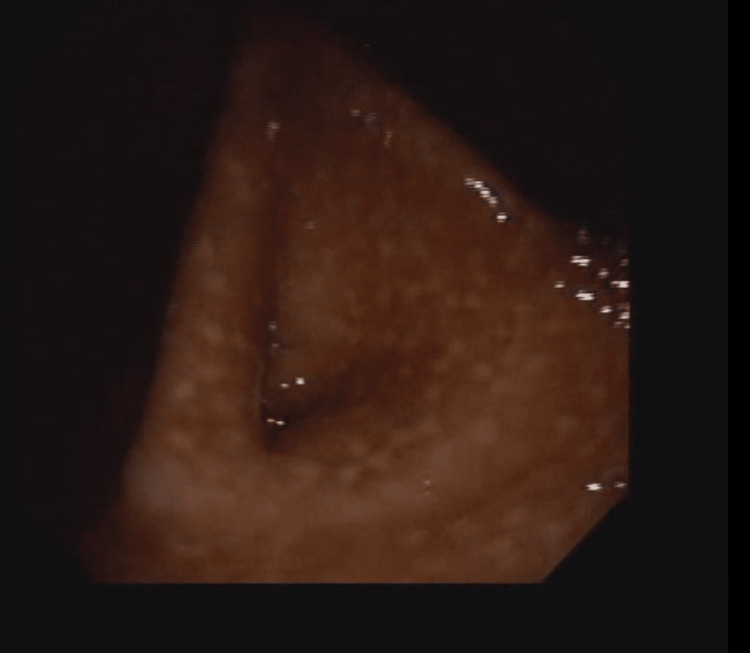
Colonoscopy image (case 2) – note: whitish patches on colonic mucosa

A 38-year-old male presented with constipation and chest pain for seven years. He was known to pass hard, pellet-like stools with associated perianal pain but no hematemesis or melaena. Over this seven-year period, he used oral bisacodyl, a stimulant laxative, once or twice a week to obtain relief from constipation. He also complained of epigastric pain, frequently radiating to the back associated with bloating, and relieved minimally by taking antacids. He also took over-the-counter antibiotics and herbal medication with no relief. He had no co-morbidities and no significant medical or surgical history. The colonoscopy showed the mucosa of the cecum appearing granular with punctuate whitish deposits (Figure 5).

The mucosae of the ascending colon, transverse colon, descending colon, sigmoid colon, and rectum/anal canal were normal. A provisional diagnosis of chronic constipation to rule out intestinal candidiasis was made. Biopsies were taken from the whitish patches on the cecal mucosa and sent for histology.

**Figure 6:**
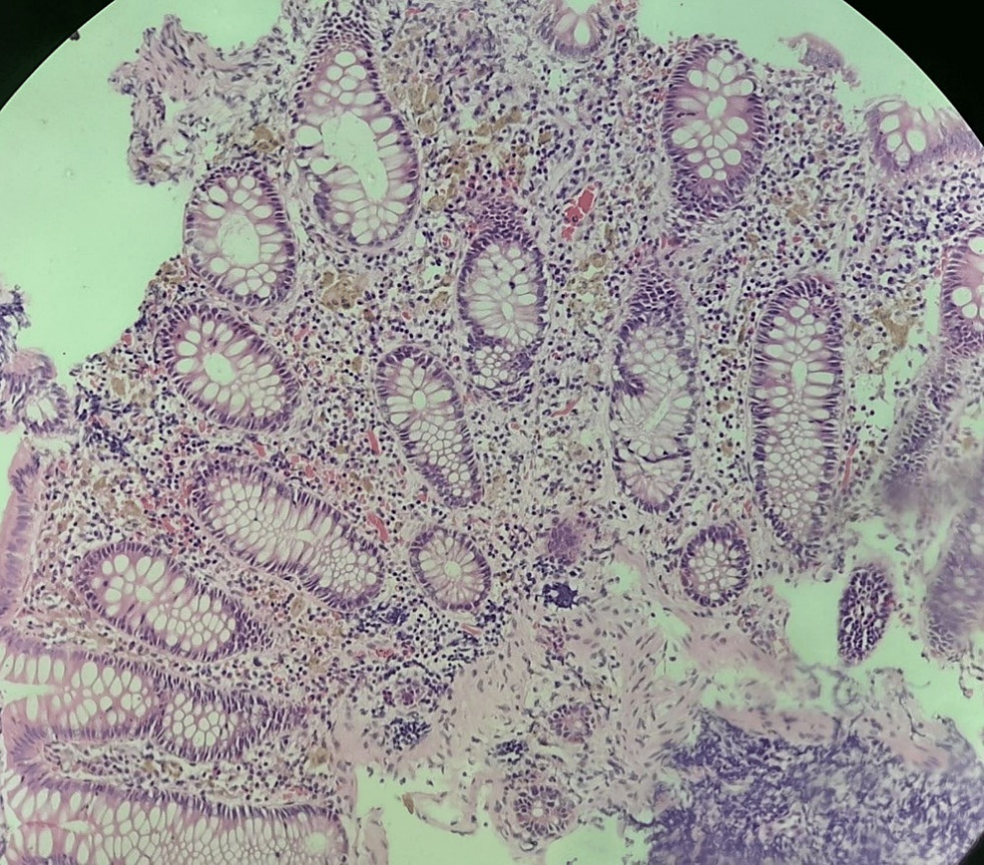
Photomicrograph case 2, H&E x20 – note: pigment laden macrophages in the lamina propria

Histology showed irregular fragments composed of colonic mucosa with unremarkable epithelium, which overlies lamina propria with quiescent goblet cell-rich glands. Numerous pigment-laden macrophages with golden-brown, granular cytoplasm were seen in the lamina propria. Mixed inflammatory infiltrates, including lymphocytes, which form occasional aggregates, plasma cells, neutrophils, and eosinophils were also seen as well as occasional foci of congested, thin-walled vascular channels. Fungal hyphae and/or spores were not seen. No atypical cells were seen (Figure 6).

**Figure 7:**
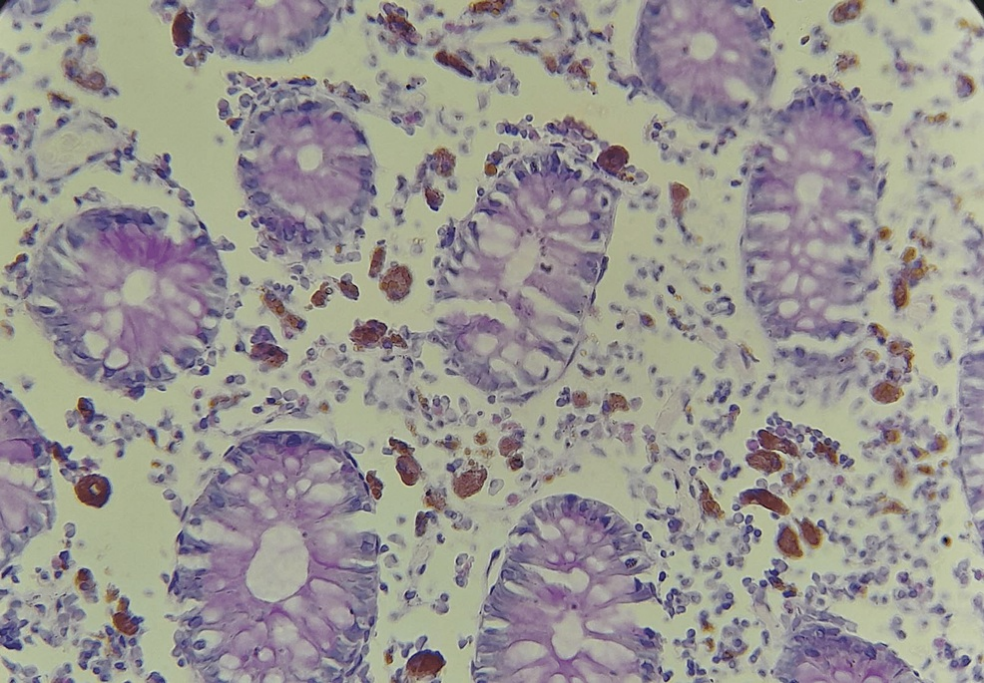
Photomicrograph Case 2, PAS x20 – Note uniform purple particles in the lamina propria macrophages PAS: periodic acid-Schiff

**Figure 8:**
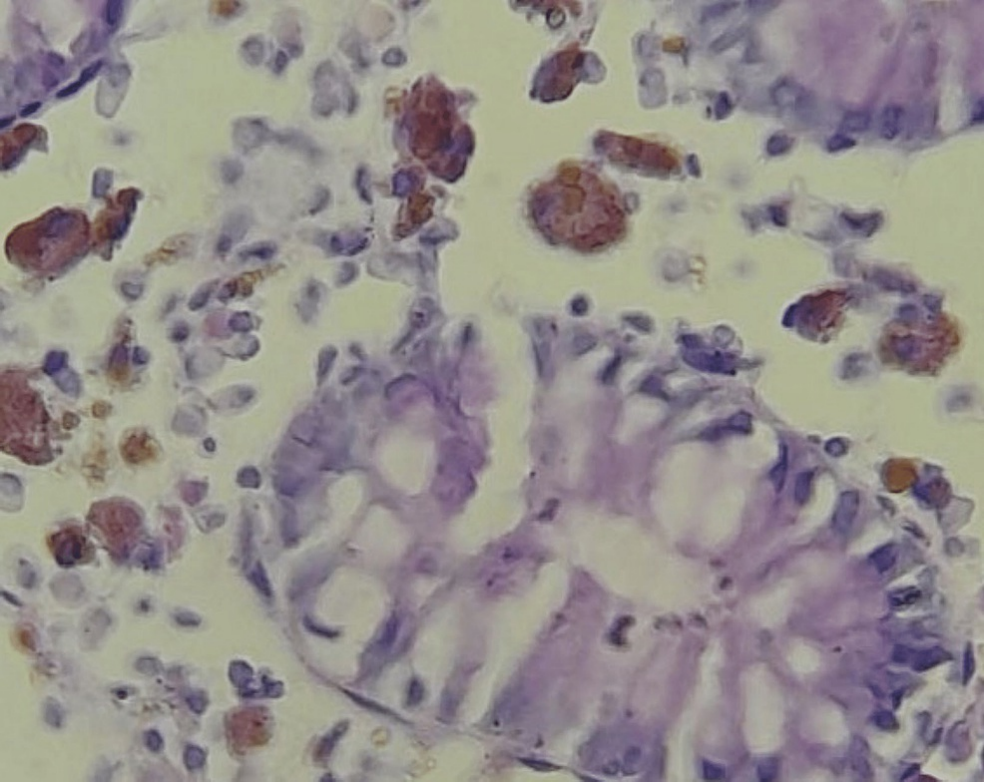
Photomicrograph case 2, PAS x100 (zoom) – note: uniform purple particles in the lamina propria macrophages PAS: periodic acid-Schiff

A diagnosis of melanosis coli was made. For confirmation, PAS histochemical and human melanoma black 45 (HMB45) immunohistochemical stains were applied to sections from the paraffin wax blocks of the specimen, which stained positive and negative, respectively (Figures 7, 8).

No treatment was given to the patient, and he has since been referred back to his primary physician after being counseled.

## Discussion

MC is a benign condition of the colon characterized by brown or black pigmentation of the lamina propria as a result of lipofuscin deposition intracellularly; and is most frequently attributed to chronic anthraquinone-based laxative use, particularly in older persons and in those with chronic constipation [1,3,8]. However, it can also be caused by prolonged use of herbal remedies and stimulant laxatives such as bisacodyl [3,10]. Both of our patients had a history of chronic constipation and prolonged bisacodyl use; and while in addition to bisacodyl, the Case 1 patient used lactulose, the Case 2 patient combined his bisacodyl with herbal remedies.

The incidence of MC is said to increase with age, and is, therefore, more common in the elderly [2,3], but both of our patients were less than 40 years old. This could be due to the length of use of laxatives being more contributory to its onset than the patient’s age. MC is not as uncommon as previously thought among Nigerians. Oluyemi AO et al. reported a prevalence of 5% in a cohort of over 750 colonoscopies performed over a 33-month period in Lagos, Nigeria [11]. Worldwide also, its incidence is rising due to the increased use of herbal remedies to manage chronic constipation [2].

The clinical features of MC are nonspecific. It is frequently an incidental finding on colonoscopy in patients with a history of chronic constipation, especially those who use anthraquinone-based laxatives over a prolonged period [1-3,5,7,10]. It may also be seen in individuals suffering from irritable bowel syndrome and other chronic inflammatory bowel diseases, without prolonged laxative use [1,9]. It is usually asymptomatic, and diagnosis is made on colonoscopy and confirmed by histology.

On colonoscopy, MC is typically noted in the large bowel, especially on the right side of the colon [3,6]. Both of our patients had right-sided lesions. The typical appearance is that of brown-black patches on the mucosa of the large intestine [1-3,5,8]. As a result of this brown-black discoloration, MC may mimic ischemic colitis in patients who present with abdominal pain [5,7]. This often presents a dilemma to surgeons who have to make a decision whether or not to resect the colon, which looks grossly ischaemic; and has led to cases of the large bowel being mistakenly resected as it was considered ischemic [5]. However, both of our patients had whitish patches on the mucosa of their colons, mimicking instead, the typical appearance of candidiasis in the esophagus [12]. To the best of our knowledge, no previous cases of whitish patches being associated with MC have been reported in the literature. Li XΑ et al. analyzed the various colonoscopy findings in MC and reported various colonic mucosal appearances, including brown, red, mucosal edema, and snakeskin, but not whitish; in all cases, the diagnosis was confirmed by histology [13].

The histologic diagnosis of MC is usually evident on light microscopy using hematoxylin and eosin (H&E) stained sections where it is characterized by the presence of golden-brown pigment-laden macrophages in the lamina propria, often termed melanized ceroid [14]. The pigment, however, is not melanin but lipofuscin, and confirmed by the presence, on light microscopy, of several uniform purple particles in the lamina propria of PAS-stained sections; and negative staining for melanin on immunohistochemistry [13]. The biopsy specimens from both our patients fulfilled these conditions hence confirming the diagnosis of MC.

It is not exactly clear why our patients’ MC manifested as whitish patches on colonoscopy but they both reported prolonged use of the stimulant laxative, bisacodyl. While the mechanism by which anthraquinone-based laxatives produce the lesions of MC has been studied and reported in the literature [2,6]; the mechanism of stimulant laxatives like bisacodyl has not been sufficiently elucidated. More studies will have to be done on patients who use stimulant laxatives like bisacodyl to determine the mechanism by which they produce MC; and whether lesions in the colonic mucosa of persons who develop it, consistently present as whitish patches on colonoscopy.

## Conclusions

MC should be considered in the differential diagnosis of color changes on colonoscopy, including whitish patches, in the colonic mucosa of patients, especially those with a history of chronic constipation and prolonged laxative use, even in the absence of the characteristic brown-black patches. Biopsies should always be taken, and the diagnosis confirmed by histology.
